# The correlation of two bone turnover markers with bone mineral density: a population-based cross-sectional study

**DOI:** 10.1186/s12891-023-06613-5

**Published:** 2023-08-24

**Authors:** Gao-Xiang Wang, Jun-Tong Li, Fang-ying Cai, Bao-Li Huang, Ze-Bin Fang, Heng-Xia Zhao, Shu-Fang Chu, De-Liang Liu, Hui-Lin Li

**Affiliations:** 1 Department of Endocrinology, Affiliated Hospital of Nanjing University of Chinese Medicine, Nanjing, Jiangsu 210029 China; 2 Department of Endocrinology, Shenzhen Traditional Chinese Medicine Hospital, Shenzhen, Guangdong 518033 China; 3 Department of Endocrinology, The Fourth Clinical Medical College of Guangzhou University of Chinese Medicine, Shenzhen, Guangdong 518033 China

**Keywords:** Bone-specific alkaline phosphatase, Bone turnover markers, Urinary N-telopeptide, Bone mineral density, NHANES

## Abstract

**Objective:**

Exploring the correlation between bone turnover marks (BTMs) with lumbar BMD in middle-aged populations.

**Methods:**

The cross-sectional analysis fetched data came from NHANES. The level of serum bone alkaline phosphatase (sBAP) and urinary N-telopeptide (uNTx) were regarded as representative of bone turnover. Lumbar BMD was the outcome of the study. Multivariable linear regression models were utilized to detect the correlation of sBAP and uNTx with Lumbar BMD.

**Results:**

The level of sBAP and uNTx was negatively correlated with lumbar BMD in every multivariable linear regression. For sBAP, this inverse correlation was stable in both men and women (*P* < 0.01). uNTx indicated a negative association after all relevant covariables were adjusted (*P* < 0.01). The men group remained the negative correlation in gender subgroup analysis (*P* < 0.01).

**Conclusion:**

This study indicated that the increased level of sBAP and uNTx associated with lumbar BMD decreased among middle-aged adults. This correlation could prompt researchers to explore further the relationship between bone turnover rate and BMD, which may provide information for the early detection of BMD loss and provide a new strategy for clinical practice.

## Introduction

Osteoporosis is a chronic, progressive, age-related skeletal disease that affects more than 200 million people globally, resulting in approximately 8.9 million fractures per year [1–3]. In the context of population aging, osteoporosis and osteoporotic fractures cause an increase in morbidity and death, resulting in a significant medical burden on society [4]. Therefore, it is essential to discover, diagnose, and treat as early as possible, which is even more critical for those middle-aged people who have reduced bone mineral density (BMD) before they reach old age. BMD is the cornerstone for the diagnosis of osteoporosis, and Dual-energy X-ray absorptiometry (DXA) is the gold standard in clinics to examine BMD. However, Multiple DXA tests in mostly asymptomatic patients are costly and a waste of medical resources [5]. Thus, increasing attention has been focused on bone turnover markers (BTMs).

Bone remodeling occurs throughout the renewing of the adult skeleton [6, 7]. The balance between bone formation and bone resorption is vital for bone remodeling [8]. BTMs reflect the activity of osteoclast (OC) cells and osteoblast (OB) cells, respectively [9]. The two common biomarkers are serum bone alkaline phosphatase (sBAP) and urinary N-telopeptide (uNTx). The level of sBAP can reflect the state of bone formation, and the level of uNTx can reflect the state of bone resorption [10]. Although BTMs have been proposed to assess osteoporosis fracture risk in the guide, recent clinical studies indicate the results are not entirely consistent [11].

**Fig. 1:**
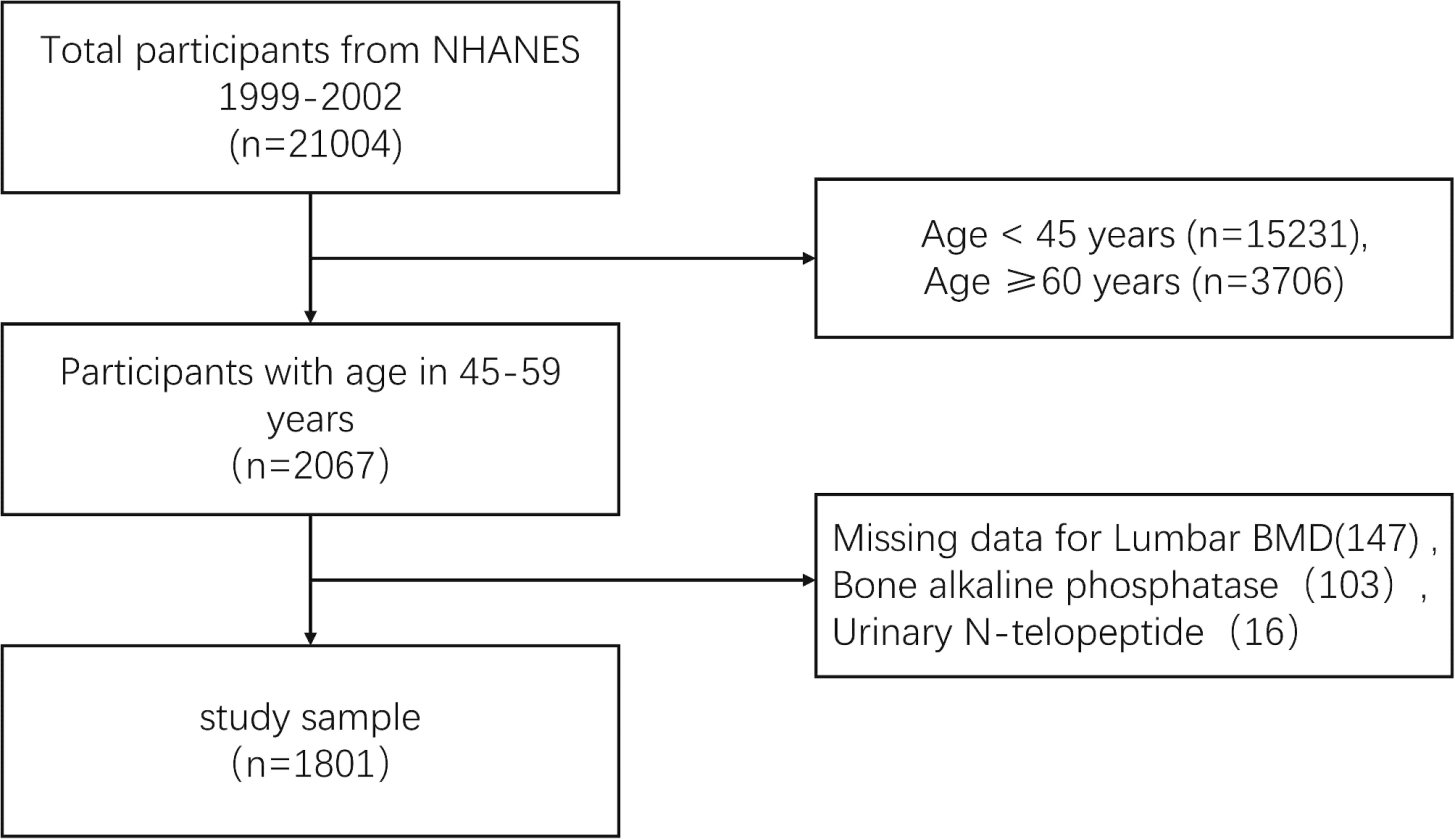
Flow chart of sample selection

BTMs are more economical and more convenient than BMD testing [12]. The changes of BTMs happen earlier than imaging changes theoretically [13]. Research showed a negative correlation between BTMs and BMD among older people recently [14]. However, whether the correlation existed in the middle-aged population is not explicit. Exploring the correlation of BTMs and BMD among the middle-aged population is beneficial for identifying patients at risk, diagnosis, getting them on treatment as early as possible, and contributing to saving medical resources. This study aims to make a full-scale evaluation of the correlation between BTMs (sBAP and uNTx) and BMD among middle-aged adults via a cross-sectional study. The analytic plan of this study has concisely shown in Fig. 1.

## Methods

### Data sources

We pooled the National Health and Nutrition Examination Survey (NHANES) data, an ongoing and sizeable cross-sectional survey in the US. The National Center for Health Statistics (NCHS) designed and launched the survey in the 1960s. The survey became an ongoing program to meet more public health and nutrition needs in 1999. NHANES aimed to develop public health policy and resolve public health issues through gathering representative samples and providing objective evidence. The program is also innovative because of physical examinations and interviews. In this study, the level of uNTx was only collected during 1999 to 2002. Hence, the NHANES 1999–2002 datasets were obtained from the NHANES website.

### Participants

The Research Ethics Review Board of NCHS approved NHANES study. Written informed consent was obtained from each participant. A total of 2067 participants were aged 45 to 59 years old. We excluded 147 participants who were missing Lumbar BMD data, 103 missing bone alkaline phosphatase data, and 16 missing Urinary N-telopeptide data. After filtrating with exclusion criteria, 1801 participants with lumbar BMD, sBAP, and uNTx data were analyzed in this study.

### BMD and BTMs

Lumbar BMD was the golden criterion to classify the status of osteoporosis and measured by DXA, which is a method recommended by WHO [15]. Access Ostase assay and Tandem-MP Ostase Immuno-Enzymetric were used to examine the activity of sBAP signifying the bone fraction. uNTx was regarded as a biomarker of bone resorption. The level of uNTx was measured via the Osteomark and Vitros ECI on-spot urine specimens [16].

### Concomitant variables

Some covariates were possible confounders in the associations between BTMs and BMD. These covariates were classified in line with a study about NHANES. Continuous potential confounders included age (in years), poverty to income ratio, body mass index, blood urea nitrogen, total protein, total cholesterol, serum uric acids, serum calcium, and serum phosphorus. Categorical possible confounders include gender (male or female), race (five categories), level of education (five categories), physical activities (five categories), smoking habits, and alcohol consumption. The acquisition processes and information about all variables in the study are freely available and detailed on the NHANES database.

### Statistical methods

All statistical analyses were performed in the study via package R (http://www.R-project.org) and Empower Stats *P* < 0.05 indicated a statistically significant level. Sampling weights were used to reveal representative data, which were civilian and noninstitutional, among the US population and achieve the goal of NHANES through implementing the analytical guideline compiled by NCHS. This study utilized a descriptive approach and developed three multivariable linear regression models,, including no concomitant variables were regulated, some concomitant variables were regulated, and all concomitant variables mentioned in Table 1 were regulated. These models and different subgroup analyses were performed to verify relevance between each variable and the level of BMD and were constructed in conformity to NHANES data. A weighted generalized additive model was used to solve the nonlinear problem, and a smooth curve was fitted.

**Table 1:** Weighted characteristics of the study sample

	Male (n = 898)	Female (n = 903)	P value
**Age (years)**	51.25 ± 4.24	51.15 ± 4.18	0.59
**Race/ethnicity (%)**			0.03
Mexican American	5.06	4.59	
Other Hispanic	6.42	6.35	
Non-Hispanic White	76.72	73.34	
Non-Hispanic Black	8.95	9.76	
Other race - including multi-racia	2.85	5.96	
**Educational level (%)**			0.38
Less than 9th grade	5.37	5.42	
9–11th grade	11.1	14.02	
High school graduate/GED or equivalent	22.18	22.11	
Some college or AA degree	30.82	30.61	
College graduate or above	30.53	27.83	
**Ratio of family income to poverty**	3.55 ± 1.50	3.47 ± 1.58	0.28
**Smoked at least 100 cigarettes in life (%)**			< 0.01
No	36.23	52.51	
Yes	63.77	47.49	
**How often drink alcohol over past 12 months**	6.38 ± 31.32	3.37 ± 6.95	< 0.01
**Average level of physical activity each day (%)**			< 0.01
Sits, not walk about very much	24.45	29.35	
Walk a lot	47.29	53.89	
Lift light load or have to climb stairs or hills often.	18.57	14.08	
Heavy activity or heavy loads.	9.69	2.69	
**Body mass index (kg/m2)**	28.64 ± 5.76	28.93 ± 7.02	0.33
**Bone alkaline phosphatase (ug/L)**	15.38 ± 6.14	14.65 ± 6.51	0.02
**Urinary N-telopeptide (umol BCE)**	0.41 ± 0.33	0.31 ± 0.30	< 0.01
**Serum calcium (mg/dL)**	9.46 ± 0.36	9.42 ± 0.41	0.03
**Serum phosphorus (mg/dL)**	3.49 ± 0.51	3.65 ± 0.56	< 0.01
**Serum uric acid (mg/dL)**	6.04 ± 1.34	4.74 ± 1.26	< 0.01
**Total cholesterol(mg/dL)**	214.84 ± 49.50	213.32 ± 37.01	0.46
**Total protein (g/dL)**	7.37 ± 0.44	7.35 ± 0.46	0.30
**Blood urea nitrogen (mg/dL)**	14.80 ± 4.26	13.48 ± 4.38	< 0.01
**Lumbar bone mineral density (g/cm2)**	1.03 ± 0.17	1.04 ± 0.16	0.41

Notes: For continuous variables, the Mean ± SD was employed and a linear regression model was used to calculate the P-value. For categorical variables, the % was employed and a chi-square test was used to calculate the *P*-value

## Results

### Participant characteristics

The weighted characteristics of participants in this study are shown in Table 1. Men smoke and drink more frequently than women; meanwhile, they get more physical activity each day. For laboratory data, the activity of sBAP and level of uNTx, serum calcium, serum uric acid, and blood urea nitrogen were markedly lower in women compared to men. However, women had a significantly higher level of serum phosphorus than men. The other variables had no significant difference between the genders.

### Associations of sBAP with lumbar BMD

There was a negative association between the activity of sBAP and the level of lumbar BMD in all three models presented in Table 2 (model 1: β=-0.004, 95% CI: -0.005 to -0.003, *P* < 0.01; model 2: β=-0.004, 95% CI: -0.005 to -0.003, *P* < 0.01; model 3: β=-0.004, 95% CI: -0.006 to -0.003, *P* < 0.01). The tendency existed significantly among four sBAP quartile subgroups (*P* < 0.01). In subgroups classified by gender, the inverse correlation still existent both in men and women with non-adjusting, adjusting selected, and adjusting all variables (*P* < 0.01). Moreover, Mexican American, White, and Black race groups presented a negative association after adjusting all variables (*P* < 0.01, *P* < 0.01, *P* < 0.05).

**Table 2:** The correlation between Lumbar bone mineral density (g/cm2) and Bone alkaline phosphatase (ug/L)

Exposure	Non-adjusted	Adjust I	Adjust II
**Bone alkaline phosphatase (ug/L)**	-0.004 (-0.005, -0.003) **	-0.004 (-0.005, -0.003) **	-0.004 (-0.006, -0.003) **
**Quintiles of Bone alkaline phosphatase**			
Lowest quartiles	reference	reference	reference
2nd	-0.016 (-0.036, 0.004)	-0.018 (-0.038, 0.002)	-0.024 (-0.044, -0.004) *
3rd	-0.033 (-0.054, -0.013) **	-0.030 (-0.051, -0.009) **	-0.042 (-0.063, -0.022) **
4th	-0.071 (-0.092, -0.049) **	-0.068 (-0.089, -0.046) **	-0.074 (-0.096, -0.052) **
*P* for trend	< 0.01	< 0.01	< 0.01
**Stratified by Sex**			
Male	-0.004 (-0.006, -0.002) **	-0.004 (-0.005, -0.002) **	-0.004 (-0.006, -0.002) **
Female	-0.005 (-0.006, -0.003) **	-0.004 (-0.006, -0.003) **	-0.004 (-0.006, -0.002) **
**Stratified by Race**			
Mexican American	-0.003 (-0.005, -0.001) **	-0.003 (-0.005, -0.001) **	-0.003 (-0.005, -0.002) **
Other Hispanic	-0.004 (-0.008, -0.001) *	-0.004 (-0.007, -0.000) *	-0.002 (-0.005, 0.001)
Non-Hispanic White	-0.005 (-0.006, -0.003) **	-0.004 (-0.006, -0.003) **	-0.005 (-0.007, -0.003)**
Non-Hispanic Black	-0.003 (-0.006, -0.001) *	-0.003 (-0.006, -0.000) *	-0.004 (-0.007, -0.001) *
Other race - including multi-racia	-0.004 (-0.009, 0.001)	-0.004 (-0.009, 0.001)	-0.005 (-0.010, 0.001)

Notes: Model 1: Model 1: No covariates were adjusted. Model 2: Age, gender, race were adjusted. Model 3: All Covariates were adjusted. In the subgroup analysis, the model is not adjusted for the stratification variable itself. * *P* < 0.05, ** *P* < 0.01

### Associations of uNTx with lumbar BMD

In three constructed models, a negative correlation existed between uNTx and Lumbar BMD (model 1: β=-0.027, 95% CI: -0.051 to -0.004, *P* < 0.05; model 2: β=-0.038, 95% CI: -0.062 to -0.015, *P* < 0.01; model 3: β=-0.037, 95% CI: -0.061 to -0.014, *P* < 0.01). In model 2 and model 3, there were significant differences in the trend of uNTx quartile subgroups. On the other hand, subgroups stratified by gender analysis indicated the negative correlation remained presented in men (β=-0.052, 95% CI: -0.084 to -0.020, *P* < 0.01). Among five-race subgroups, only the non-Hispanic white group (β=-0.044, 95% CI: -0.079 to -0.010, *P* < 0.05) still showed the negative association (Table 3).

**Table 3:** The correlation between Lumbar bone mineral density (g/cm2) and Urinary N-telopeptide (umol BCE)

Exposure	Non-adjusted	Adjust I	Adjust II
Urinary N-telopeptide (umol BCE)	-0.027 (-0.051, -0.004)*	-0.038 (-0.062, -0.015)**	-0.037 (-0.061, -0.014)**
Quintiles of Urinary N-telopeptide			
Lowest quartiles	reference	reference	reference
2nd	0.011 (-0.009, 0.032)	0.008 (-0.012, 0.028)	0.001 (-0.019, 0.021)
3rd	0.009 (-0.012, 0.031)	0.002 (-0.019, 0.023)	-0.009 (-0.030, 0.012)
4th	-0.019 (-0.040, 0.003)	-0.027 (-0.049, -0.006)*	-0.031 (-0.053, -0.010)**
P for trend	0.12	0.02	< 0.01
Stratified by Sex			
Male	-0.048 (-0.081, -0.015)**	-0.058 (-0.091, -0.025)**	-0.052 (-0.084, -0.020)**
Female	-0.001 (-0.036, 0.033)	-0.011 (-0.046, 0.023)	-0.008 (-0.043, 0.026)
Stratified by Race			
Mexican American	-0.032 (-0.075, 0.011)	-0.040 (-0.083, 0.003)	-0.039 (-0.081, 0.003)
Other Hispanic	-0.057 (-0.144, 0.030)	-0.068 (-0.154, 0.017)	-0.070 (-0.155, 0.015)
Non-Hispanic White	-0.048 (-0.082, -0.015)**	-0.052 (-0.087, -0.018)**	-0.044 (-0.079, -0.010)*
Non-Hispanic Black	0.010 (-0.039, 0.058)	0.010 (-0.038, 0.059)	0.010 (-0.040, 0.061)
Other race - including multi-racia	0.071 (-0.077, 0.220)	0.086 (-0.064, 0.236)	0.025 (-0.136, 0.186)

Notes: Model 1: No covariates were adjusted. Model 2: Age, gender, race were adjusted. Model 3: All Covariates were adjusted. In the subgroup analysis, the model is not adjusted for the stratification variable itself. * *P* < 0.05, ** *P* < 0.01

**Fig. 2:**
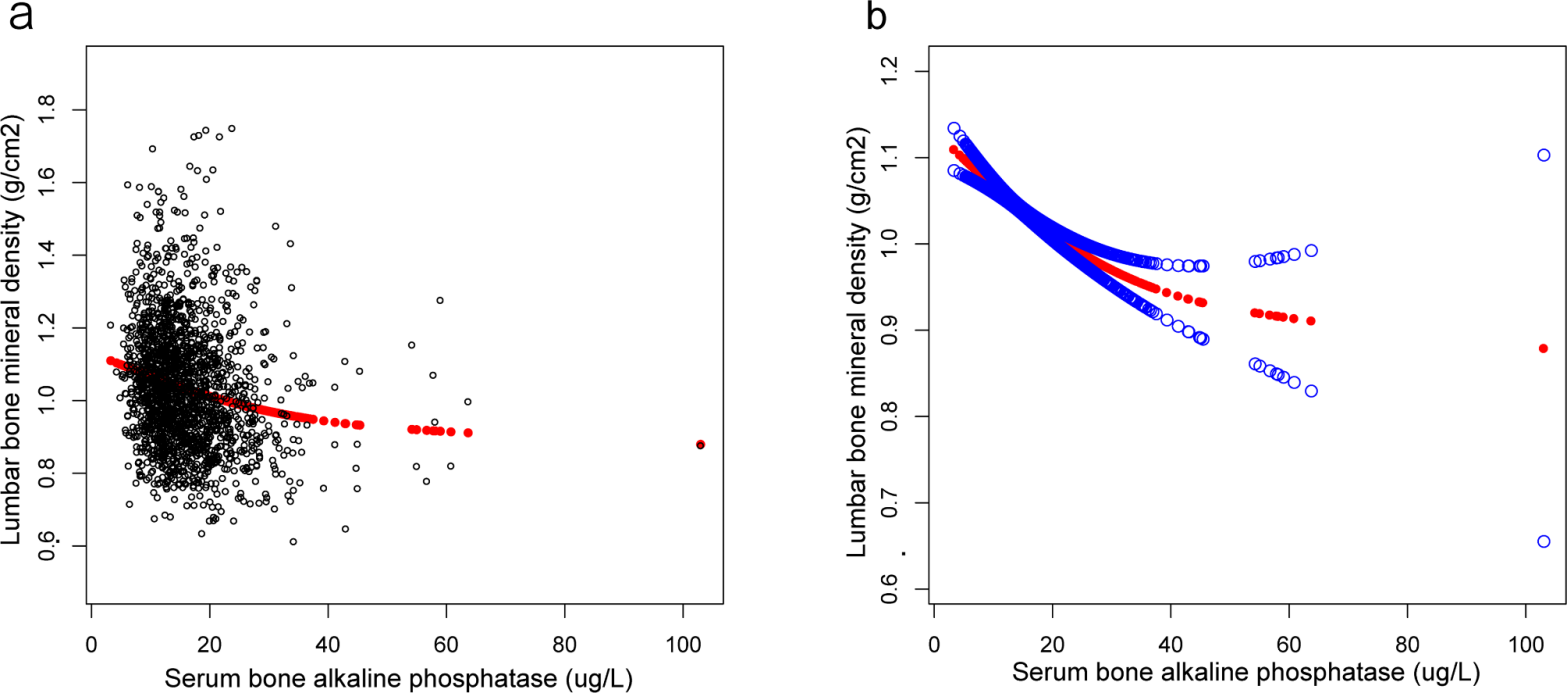
The correlation between lumbar bone mineral density and serum bone-specific alkaline phosphatase. **(a)** Each black point represents a sample. **(b)** The solid red line represents the smoothed curve that fits the variables. The blue bands represent the 95% confidence interval for the fit

**Fig. 3:**
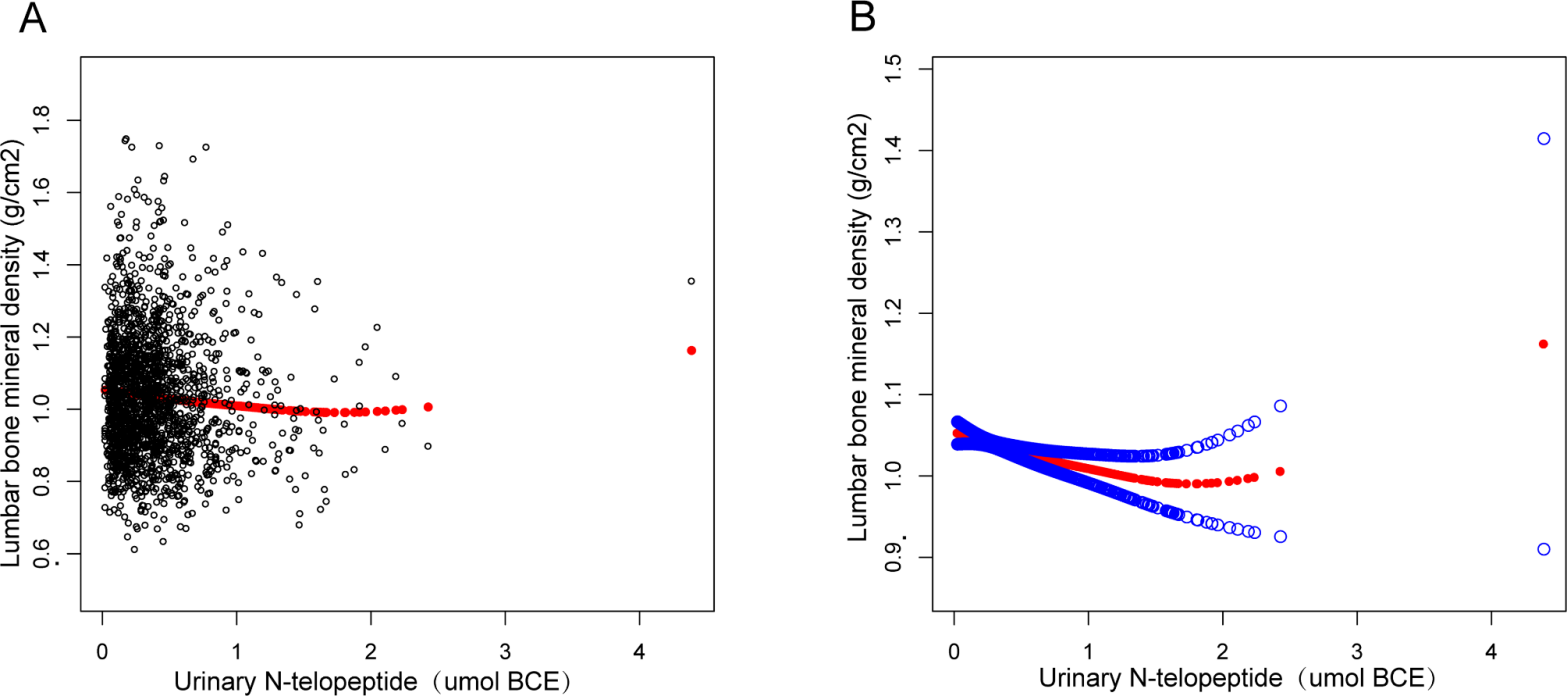
The correlation between lumbar bone mineral density and urinary N-telopeptide. **(a)** Each black point represents a sample. **(b)** The solid red line represents the smoothed curve that fits the variables. The blue bands represent the 95% confidence interval for the fit

**Fig. 4:**
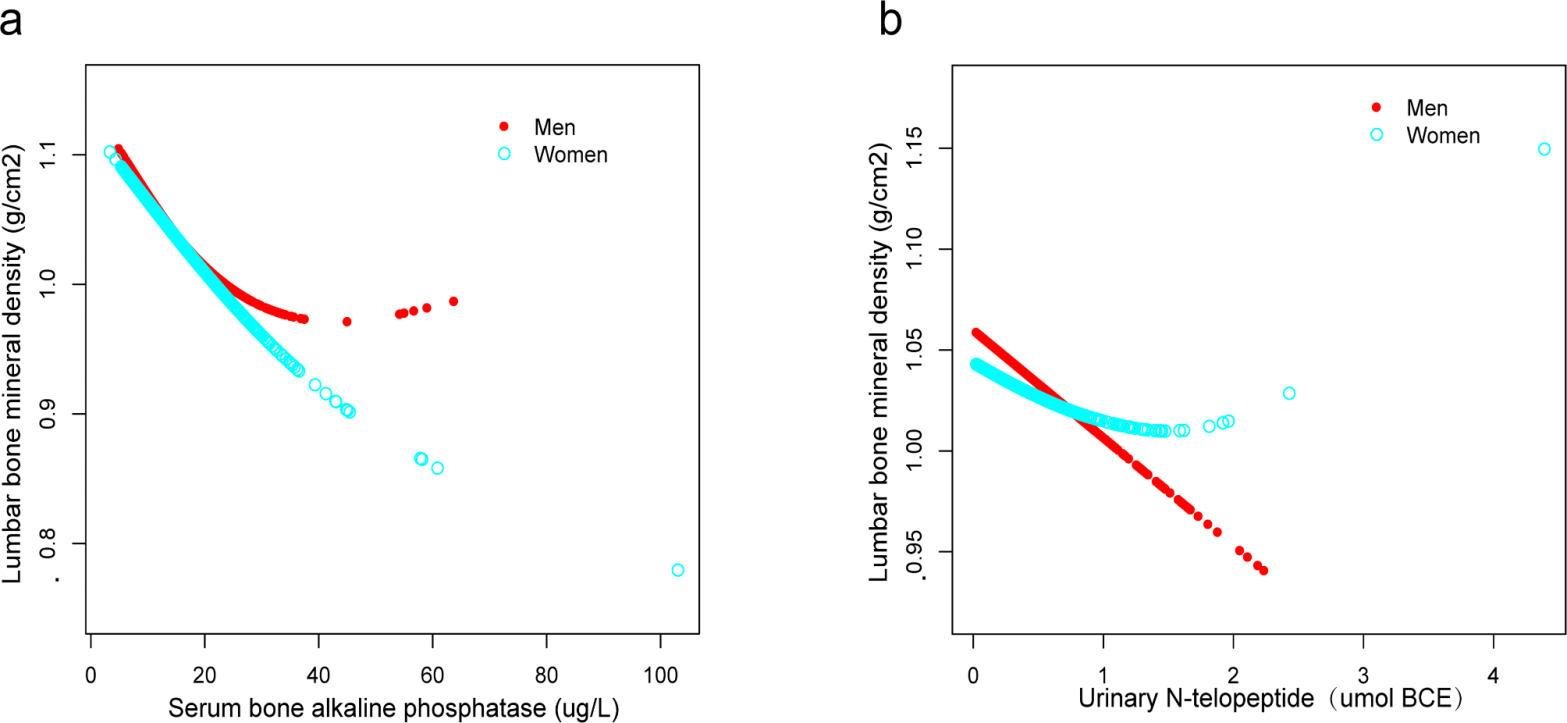
The correlation between serum bone-specific alkaline phosphatase, urinary N-telopeptide, and lumbar bone mineral density

Furthermore, this study adopted weighted generalized additive models and fitted smoothing curves to assess individuals and non-linear relations between Lumbar BMD and sBAP or Lumbar BMD and uNTx, respectively. These results mentioned above were verified in Figs. 2, 3 and 4. Hence, the study revealed a negative association between Lumbar BMD, sBAP, and uNTx.

## Discussion

Osteoporosis is an age-related disease that impacts the quality of life and even increases disability. In China, the appraisal of osteoporosis prevalence was 13%, and there will be 4.83 million patients with osteoporosis in 2035 [17, 18]. Moreover, patients do not know they are osteoporosis until a fracture. Thus, early prevention and diagnosis are crucial for managing and treating osteoporosis. The purpose of the present study was to investigate the correlation between BTMs (sBAP and uNTx) with lumbar BMD in a large and representative specimen among the middle-aged population in US. In brief, our results revealed that participants with increased sBAP and uNTx accompanied a decreased lumbar BMD among middle-aged adults. Furthermore, we verified that the trend of non-linear correlation between BTMs (sBAP and uNTx) with lumbar BMD was in accord with multivariable linear regressions. This correlation may prompt researchers to investigate further the relationship between bone turnover rate and BMD.

BTMs could reflect the bone turnover rate dynamically and guide the diagnosis and treatment in clinical practice. The Fracture Intervention Trial manifested the decreased rate of bone turnover had a strong correlation with reductions of fractures in women with alendronate treatment [19]. Thus, the bone resorption and formation biomarkers have close associations with bone mass loss and the incidence of fractures.

sBAP is an indicator commonly used in accessing the activity of OB cells. In this study, multivariable linear regression models revealed that the level of sBAP was negatively correlated with BMD. Our results are consistent with previous clinical research evidence. The link between BTMs and BMD both in middle-aged and elderly individuals indicates a negative correlation and can be broadly utilized in clinical settings. Serum sBAP was reported to be associated inversely with BMD at baseline, 12 months, and 24 months in chronic kidney disease patients [20]. Furthermore, 94 women who had entered menopause were separated into three groups (normal BMD, osteopenia, and osteoporosis) to detect investigate the correlations between BTMs and BMD. Results indicated only sBAP and CTX showed significant correlations with BMD [21]. Nevertheless, in 115 axial spondyloarthritis (SpA) patients, there were no significant correlations between the increased sBAP level with abnormal structural damage and low BMD in subgroup analysis [22]. But both in men and women subgroups of our results, the inverse association remained. The specificity of the included participants probably caused the inconsistency.

For evaluating the state of bone resorption, u-NTx showed fast and significant change with increased bone turnover rate and indicated a stronger correlation with BMD than other biomarkers. A multicenter, community-based study revealed that detection of u-NTx was beneficial to identify women suffering from fast bone loss in the early postmenopause [23]. A retrospective evaluation uncovered u-NTx was an early detection marker of poor compliance or secondary osteoporosis [24]. In a word, the clinical application value of u-NTx has been confirmed. The study also found that the u-NTx level is negatively associated with BMD. However, the subgroups analysis revealed that only the man subgroup still had an inverse correlation with BMD.

In conclusion, this study indicated sBAP and u-NTx were negatively correlated with BMD, and reduction of bone turnover was healthy for the skeleton. Despite mentioned above, there were some limitations in the present study. First, more prospective observation research should explore the deeper connection between sBAP and u-NTx with lumbar BMD. Second, we selected the urine sample instead of the serum sample for detecting the level of NTx. But the serum sample lacked tangible proof that it was preferable to the urine sample. Third, BMD levels measured by DXA still have some technical and methodological difficulties. Improve diagnosis and treatment capability is beneficial for accessing the association between BTMs with BMD.

## Data Availability

The survey data is openly available on the internet (www.cdc.gov/nchs/nhanes/) for data users all around the world.
